# Supplementary material for: Comparative genomics provides new insights into the diversity, physiology, and sexuality of the only industrially exploited tremellomycete: Phaffia rhodozyma
Source: BMC Genomics. 2016 Nov 9;17:901. doi: 10.1186/s12864-016-3244-7 (PMC5103461; doi:10.1186/s12864-016-3244-7)
Supplement: Additional file 6: — List of orphan genes with links to PFAM (related to Additional file 1: Table S1). (ZIP 1428 kb) [file 12864_2016_3244_MOESM6_ESM.zip › PFAM_UNIQUE/G03980_P.html]

#                                                                            --- full sequence --- -------------- this domain -------------   hmm coord   ali coord   env coord
  
# target name        accession   tlen query name           accession   qlen   E-value  score  bias   #  of  c-Evalue  i-Evalue  score  bias  from    to  from    to  from    to  acc description of target
  
#------------------- ---------- ----- -------------------- ---------- ----- --------- ------ ----- --- --- --------- --------- ------ ----- ----- ----- ----- ----- ----- ----- ---- ---------------------
  
G03980\_P             -            283 Apc1                 PF12859.2    105       1.1    8.2   8.3   1   2    0.0021       4.2    6.3   1.5    57    94   104   144    70   148 0.63 - Anaphase-promoting complex subunit 1  
G03980\_P             -            283 Apc1                 PF12859.2    105       1.1    8.2   8.3   2   2    0.0032       6.3    5.7   0.7    42    97   201   255   166   262 0.67 - Anaphase-promoting complex subunit 1  
G03980\_P             -            283 DUF1908              PF08926.6    282     0.046   11.1   6.1   1   1   7.3e-06     0.022   12.2   4.3    19   148    91   226    73   240 0.69 - Domain of unknown function (DUF1908)
